# Delivering Degradation: Nanomedicine and Programmable Proximity Platforms for Targeted Protein Degradation

**DOI:** 10.3390/pharmaceutics18091097

**Published:** 2026-08-31

**Authors:** Adnan Amin, Touseef Nawaz, Oberdan Oliveira Ferreira, Mozaniel Santana de Oliveira

**Affiliations:** 1 Department of Life Sciences, Yeungnam University, Gyeongsan 38541, Republic of Korea; 2 Department of Pharmacy, Qurtuba University of Science and Information Technology, Peshawar 25100, Pakistan; touseefktk@qurtuba.edu.pk; 3 Program in Biotechnology, Federal University of Pará, Belém 66075-110, Brazil; oberdan@museu-goeldi.br; 4 Institute of Health Sciences, Federal University of Pará, Belém 66075-110, Brazil; mozaniel@ufpa.br

**Keywords:** targeted protein degradation, proteolysis-targeting chimeras, nanomedicine, aptamer-based degradation, microRNA

## Abstract

Targeted protein degradation (TPD) represents a whole new paradigm in cell-level therapeutic design, with its ability to remove target proteins, normally through the endogenous proteasomal, lysosomal, or autophagic systems, rather than the traditional occupancy-driven inhibition approach. But the clinical efficacy of degraders is becoming more restricted based on delivery rather than efficacy only. Many proteolysis-targeting chimeras and new proximity-inducing systems have low solubility, are impermeable, are pharmacodynamically complicated, lack tissue selectivity, and cannot fully access the intracellular space. Nanomedicine and PD platforms could provide strategies not only to overcome these challenges, but also to provide other advantages, including enhancing exposure to degraders, biodistribution, controlled release, and context-dependent activation. This critical review is an outline of all lipid, polymeric, inorganic, biomimetic, targeted, activatable, and self-assembling delivery systems for TPD. We assess compositional considerations, in vitro and in vivo evidence, challenges for translation, and clinical endpoints required to support delivery-enabled degradation. Trusted TPD therapeutics need to relate different aspects of their design, such as degrader chemistry, carrier structure, disease biology, and pharmacodynamic biomarkers, to one another. Further investigations are needed to establish intact delivery of the degrader to the target, target depletion in relevant tissues, prolonged pharmacodynamics, favorable safety, and compelling therapeutic benefit relative to free degraders or traditional inhibitors. Thus, it is important to view delivery not simply as an additional step during formulation but as a design principle necessary for the reliable clinical outcome of degradation medicine.

## 1. Introduction

A shift in therapeutic design from inhibition of protein function to the elimination of disease-relevant proteins is termed Targeted Protein Degradation (TPD) [1]. Occupancy-driven pharmacology involves a typical mechanism of action, in which, as an enzyme or a receptor, a target must be bound continuously by traditional small-molecule therapeutics to block its activity [2]. TPD instead relies on event-driven pharmacology in which a degrader brings into proximity a protein of interest and the cellular degradation machinery to trigger protein removal instead of just transient functional inhibition [3]. This distinction can provide TPD with a conceptual edge over targets lacking conventional pockets, function as scaffolding, or exert disease activity via nonenzymatic processes [4].

PROTACs are heterobifunctional molecules that bring a protein of interest into proximity with an E3 ubiquitin ligase, thereby promoting target ubiquitination and proteasomal degradation [5]. Molecular glue degraders are small molecules that induce or stabilize an interaction between an E3 ligase and a neo-substrate, resulting in substrate ubiquitination and degradation [6]. The scope of targeted degradation also extends beyond the ubiquitin–proteasome system. Lysosome-targeting chimeras (LYTACs) engage cell-surface lysosome-trafficking receptors to direct extracellular or membrane-associated proteins to lysosomes [7]; whereas, antibody-based PROTACs (AbTACs) recruit transmembrane E3 ligases to promote the internalization and lysosomal degradation of cell-surface proteins [8]. Autophagy-targeting chimeras (AUTACs) use target-binding and autophagy-inducing components to promote selective autophagic clearance [9]; whereas, autophagosome-tethering compounds (ATTECs) connect specific targets to the autophagy machinery for lysosomal degradation [10]. Together, these modalities extend induced-proximity degradation across proteasomal, endolysosomal, and autophagic pathways.

Although TPD is an attractive mechanistic approach, the technique does reveal a fundamental translation challenge: precedent potent biochemical or cellular degradation does not imply that the drug would perform as efficiently from a therapeutic standpoint in vivo [11]. Many degraders have a high molecular weight and polarity, poor aqueous solubility and membrane permeability, and complicated pharmacokinetics [12]. These features can limit access to tissues and/or cells, escape to endosomes, and the efficient formation of a ternary complex needed for degradation [13]. Such restrictions are not a peripheral matter of formulation, but rather constitute the criteria for deciding if a degrader can reach the right tissue, enter the correct cell, access the appropriate cell compartment, and utilize the apt cellular degradation machinery.

Therefore, nanomedicine has been turned into a TPD development strategy [14]. Using nanocarriers to deliver a degrader can enhance payload solubility, shield chemically labile compounds, alter biodistribution, lower systemic exposure, and provide ligand-, microenvironment-, or stimulus-dependent payload release. Lipid, polymeric, inorganic, biomimetic, and hybrid nanoplatforms have been employed to deliver PROTACs and related degraders [15,16]. Therefore, delivery systems should be measured not only based on conventional nanomedicine metrics but also include degradation-specific metrics. Unless they provide evidence of a measurable depletion of target cells within the tissue at tolerable doses, accumulation of tumors, cellular uptake, or payload release, their applicability is inadequate [17].

The delivery challenge is multiplied with programmable proximity platforms. Triggered/logic-gated degraders are intended to pinpoint proteolysis to specific biological contexts—e.g., tumor microenvironment, disease-associated enzymes, nucleic acid signatures, etc., and/or external stimuli [18]. Spatiotemporal control of degrader activation can be achieved via linker engineering, cleavable motifs, photoswitchable designs, and responsive assemblies [19]. While compositional complexity, analytical burden, and regulatory uncertainty related to these degradation types are increased, so is the possibility of reduced off-target degradation [20].

This critical narrative review focuses principally on original studies in which PROTACs, pro-PROTACs, or PROTAC-forming precursors are incorporated into, conjugated to, or assembled as nanoscale delivery systems. General nanomedicine evidence is considered only where it directly informs delivery barriers relevant to PROTACs, including formulation stability, membrane translocation, intracellular trafficking, endosomal escape, pharmacokinetics, biodistribution, and safety. The review further distinguishes experimentally demonstrated nano-PROTAC performance from mechanisms inferred from broader nanomedicine research. Its principal contribution is the integrated comparison of delivery mechanism, quantitative degradation performance, translational maturity, and methodological limitations across emerging nano-enabled PROTAC platforms.

This mini-review critically evaluates delivery as a major determinant of the translational performance of targeted protein degraders. The next step in advancement involves increasing focus on an integrated design comprising degraders, carriers, triggers, pharmacokinetics, and pharmacodynamic biomarkers. In addition to binding its target, a clinically relevant degrader needs to reach the right biological compartment, turn on at the appropriate time, trigger enough target degradation, and be exposed within a safe window.

## 2. Literature Search Strategy

This narrative review was based on a targeted search of PubMed/MEDLINE, Scopus, Web of Science, ScienceDirect, Google Scholar, and Consensus. English-language, peer-reviewed studies published between 2020 and 2026 were prioritized, while earlier foundational studies were included for mechanistic context when necessary. Search terms included “targeted protein degradation,” “PROTAC,” “molecular glue,” “LYTAC,” “AUTAC,” “ATTEC,” “nano-PROTAC,” “polymeric nanoparticles,” “inorganic nanocarriers,” “biomimetic vesicles,” “aptamer-based degradation,” “activatable PROTACs,” “intracellular trafficking,” “pharmacokinetics,” “biodistribution,” “toxicity,” and “clinical translation.” Studies were included if they addressed degrader delivery, carrier design, intracellular release, target degradation, pharmacokinetics, biodistribution, safety, programmable activation, manufacturing, or translational relevance. Primary experimental studies were given precedence; recent reviews were included for broader mechanistic and regulatory context. Preprints, conference abstracts without full publications, dissertations, retracted studies, and unrelated reports were excluded. Because this review was designed to evaluate mechanistic and translational evidence rather than patterns of scholarly output, bibliometric indicators were not used as proxies for the biological or clinical importance of delivery. Recent independent scientometric analyses identifying nanotechnology-based delivery, oral bioavailability, tumor-targeted delivery, and central nervous system penetration as emerging TPD research fronts [21,22] provide complementary field-level context; whereas, the conclusions of this review are derived from the empirical evidence evaluated in the subsequent sections.

## 3. Compositional and Characterization Considerations

Formulation stability is one of the factors influencing the composition of a TPD delivery system [23]. As such, nothing less than characterization tools able to relate the properties of the material to degradation-associated pharmacology must be employed for nano-enabled TPD systems, and not for conventional nanomedicine [24].

### 3.1. Physicochemical Liabilities of Degraders

The space of several proteolysis-targeting chimeras (PROTACs) is beyond the traditional definition of a small-molecule drug. They are bifunctional in nature, which often enhances their molecular weight, polar surface area, hydrogen bonding capability, conformational flexibility, and lipophilicity [25]. These properties can lower aqueous solubility, passive permeability, oral absorption, and predictability in drug pharmacokinetics [26]. The two primary issues of membrane permeability and water solubility are obstacles in PROTAC translation [27]. These liabilities are not only kinetic in nature; they can also interfere with the delivery of intracellular degraders, thereby achieving enough exposure, and can suppress productive ternary complex formation [28].

Chemical stability needs specific attention; degraders may contain linkers, ligands, conjugation handles, or prodrug motifs that are susceptible to hydrolysis, oxidation, enzymatic cleavage, or premature deconjugation [29]. A delivery platform that increases the apparent solubility of the degrader would render it unstable and prevent therapeutic translation. Hence, characterization must confirm cargo, chemical integrity within the formulation, during storage, in serum, and in cell-mimicking environments.

### 3.2. Nanocarrier Composition

Various nano-enabled TPD systems can be classified into lipid, polymeric, inorganic, biomimetic, and hybrid platforms [30]. Lipid-based types can solubilize hydrophobic degraders, improve circulation behavior, and enable prodrug incorporation [31]. For instance, the physicochemical and pharmacokinetic attributes of PROTAC MZ1 were redesigned using lipid nanodisks, which enhanced in vivo tumor retention and therapeutic efficacy [27]. Polymeric nanoparticles can be engineered to degrade, ligate, and possess other controlled release properties. Long-term fate and toxicity of inorganic carriers that are employed for structural, imaging, or stimulus-responsive release must be carefully assessed. Membrane-coated nanoparticles and vesicle-like carriers may increase immune system evasion and improve cell targeting but add compositional variability to the system [32]. Hybrid systems integrate these attributes, but with added complexity can reduce reproducibility and regulatory clarity [33].

These composition–function relationships are demonstrated directly in primary nano-PROTAC studies. Lipid nanodiscs have been used to remodel the pharmacokinetic properties of an MZ1 prodrug; whereas, polymeric systems include bioorthogonal POLY-PROTAC nanoparticles and glutathione-responsive poly (disulfide amide) nanoparticles carrying ARV-771. Inorganic constructs include gold-nanocluster hybrid PROTACs targeting HER2 and carbon-dot-based PROTACs targeting PD-L1 [27,34,35]. These studies demonstrate that carrier composition influences not only solubilization and tissue exposure but also intracellular release, target accessibility, and degradation pharmacodynamics.

### 3.3. PROTAC Loading Strategies

Incorporation of PROTACs into nanocarriers includes physical-enclosure or chemical-conjugation, assembly as a prodrug, and carrier-free nanoassembly [36]. Encapsulation is straightforward and could enhance payload solubility, although its weak retention by the capsules could result in premature leakage and exposure to the system. Chemical conjugation can help optimize payload stability and association with the carrier; however, conjugation can change payload release, ternary complex formation, or E3 ligase recruitment [37]. To improve circulation or stimulus-responsive release, prodrug assembly can be achieved; however, the released species must be validated as an active degrader and not an inactive species in such a situation [38]. Interest in carrier-free nanoassembly stems from an ability to reduce excipient burden and increase the drug-loading amount; however, careful assessment of colloidal stability, reproducible assembly, and predictable disassembly are first necessary [39]. Self-assembly can also constitute a functional component of degrader activity rather than merely a loading strategy. In situ peptide assembly has been used to generate nano-PROTACs with reduced susceptibility to the hook effect, while PSMA-responsive intracellular assembly has enabled simultaneous degradation of AR, AR-V7, and HSP90 [40,41]. Nevertheless, the identity, stability, and degradation competence of the assembled species must be demonstrated under biologically relevant conditions.

Loading efficiency is not enough with TPD. The key issue is whether the loading strategy maintains degradative ability following release [42]. Hence, studies on well-formulated products should measure the amount of degrader released intact and correlate release processes to depletion of intracellular targets, in addition to cumulative drug release.

### 3.4. Critical Characterization Parameters

Characterization of nanomedicine is still pivotal as a standard procedure. Particle size, polydispersity index, zeta potential, and morphology must be reported along with encapsulation efficiency, drug loading, release kinetics, serum stability, storage stability, and colloidal stability [43]. Reviews on lipid nanoparticles underscore the importance of composition and physicochemical properties on liposome characteristics in terms of drug loading, release, stability, and in vivo performance [44]. These parameters must be expanded for degrader delivery while maintaining its integrity, percentage of active payload, protein corona effects, endosomal escape potential, intracellular release, and degradation-related pharmacodynamics [45,46].

An often criticized trait of nano-TPD studies is that they rely heavily on formulation metrics and lack degradation-specific validation [47]. Any minimal characterization should therefore analyze the potential for target(s) degradation, maximum degradation, associated kinetics, washout, and proteome-level selectivity if possible.

### 3.5. Characterizing Programmable Systems

Programmable proximity systems add another level of analytical complexity. Characterization of trigger-responsive degraders, split-degrader assemblies, logic-gated systems, and reversible platforms for trigger threshold, activation kinetics, spatial control, reversibility, and off-state leakage [48]. Cleavable, photoswitchable, flexible, and functionalized linkers can be crucial for modulating stability, pharmacokinetics, selectivity, and spatiotemporal control, and linker design is thus at the core of all considerations [19]. But cleavage chemistry by itself is not a measure of responsiveness. A programmable degrader has to be adaptable enough to display target depletion in a biologically relevant context as a functional trigger [49].

Logic-gated systems are subject to particularly tight controls. Investigators are advised to compare inactive precursors, activated degraders, triggers alone, carriers only, and non-targeted analogs [50,51]. Reversibility must be evaluated based on degradation shutdown, target resynthesis, and functional recovery.

**Figure 1:**
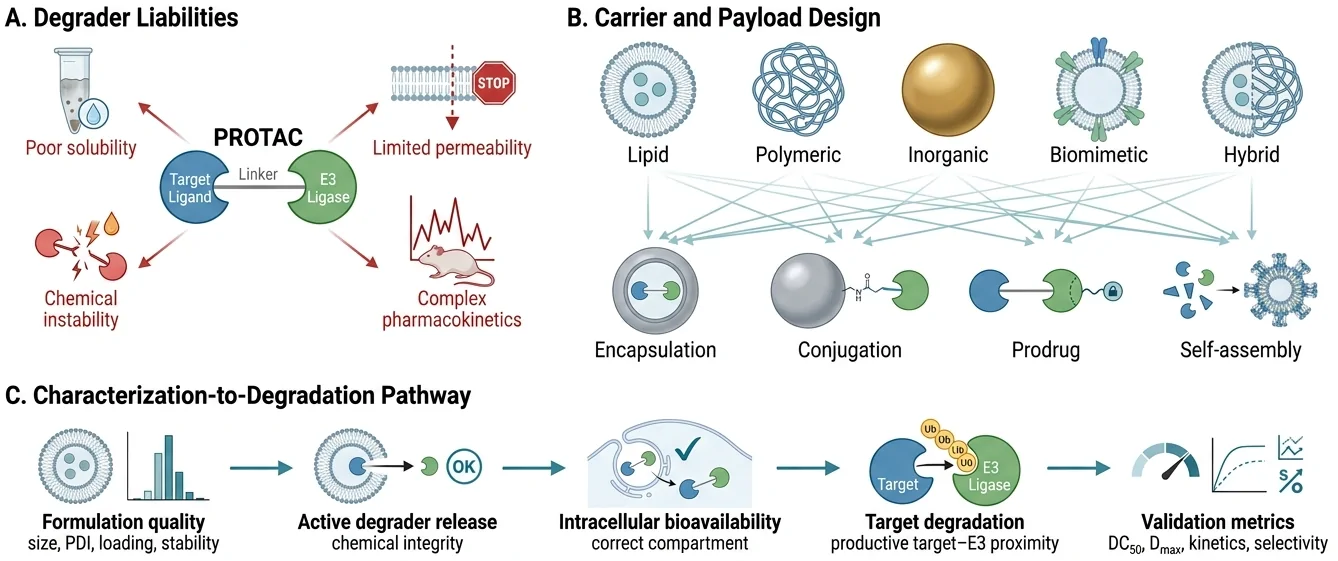
Integrated design and validation framework for nanomedicine-enabled targeted protein degradation. (**A**) Major degrader liabilities limiting intracellular exposure. (**B**) Principal carrier classes and payload-integration strategies. (**C**) Validation from formulation quality and active degrader release to intracellular bioavailability, target degradation, and assessment using DC_50_, Dmax, kinetics, and selectivity. The inset shows programmable OFF–ON activation, leakage, and reversibility.

Overall, “can the carrier load the degrader?” should be changed to “does the system provide delivery of intact active degrader to correct biological compartment and controllable and selective degradation?” This change is critical to realizing nano-enabled TPD as a translation drug delivery platform (Figure 1).

## 4. Mechanistic In Vitro Insights

A delivery system should not only degrade, but must also determine the reasons why a delivery system has to improve in vitro degradation.

### 4.1. Cellular Uptake and Endosomal Escape as Determinants of Degradation Efficiency

Entry into cells, but not breakdown into products, is required for productivity. Depending on the particle size, surface chemistry, and cell type, nanocarriers might be internalized by cells via endocytosis involving clathrin, caveolae, macropinocytosis, and/or phagocytosis [52,53]. Yet a large number of internalized particles get stuck in endosomes or lysosomes [54]. These sequestrations by proteasome-directed degraders, including PROTACs, can decrease not only the accessibility of targets to the cytosol or nucleus but also degradation despite elevated levels of cell entry [54,55].

Hence, in vitro evaluation should be able to differentiate between total uptake and bioavailable intracellular degrader [56]. Such a distinction can be supported by confocal microscopy, flow cytometry, subcellular fractionation, and endosomal escape experiments [54] the final deciding point remains the loss of targets from the respective endosome. Intracellular delivery and bioavailability are the two major challenges that nanotechnology has to overcome for clinical progress, as noted in reviews on TPD [55].

### 4.2. Visualization and Quantitative Assessment of Intracellular Delivery

Validation of nano-enabled targeted protein degradation requires clear differentiation among cellular uptake, endosomal membrane disruption, cytosolic or nuclear delivery of intact degrader, and functional target depletion. Super-resolution and live-cell microscopy can track labeled carriers or payloads together with endosomal markers such as EEA1, Rab5, Rab7, CD63, and LAMP1, thereby resolving carrier disassembly, vesicular trafficking, and payload redistribution at the single-vesicle level [57,58]. Galectin-8 or galectin-9 recruitment can sensitively identify endosomal membrane damage; however, galectin-positive vesicles do not necessarily release therapeutically meaningful amounts of cargo [59,60]. Recent live-cell and super-resolution imaging demonstrated that only a small fraction of RNA cargo escaped from damaged endosomes and that some disrupted vesicles contained no detectable payload [61]. Membrane damage should therefore not be treated as equivalent to productive degrader release.

Complementation-based biosensors, including Split Luciferase Endosomal Escape Quantification, provide sensitive measurements of cytosolic cargo arrival and can distinguish endosomal escape from total cellular uptake [62]. Flow-cytometric assays based on fluorescence redistribution, pulse-width changes, dye dequenching, or reporter complementation enable higher-throughput comparison of formulations and cellular subpopulations [63]. Nevertheless, these methods may require cargo modification or engineered reporter cells and generally provide limited spatial information. Their results should be confirmed by microscopy and appropriate controls for extracellular fluorescence, membrane-associated material, nonspecific membrane disruption, and cytotoxicity.

Subcellular fractionation coupled with targeted LC–MS/MS provides a complementary label-free approach for quantifying chemically intact degrader in cytosolic, membrane/organelle, and nuclear fractions [64]. Fraction purity, analyte recovery, extracellular carryover, matrix effects, chemical stability, and cross-contamination must be controlled. Moreover, LC–MS/MS generally measures total compartment-associated degrader rather than its unbound, pharmacologically available concentration. Single-cell proteomics can further reveal heterogeneity in target abundance, E3-ligase expression, target depletion, pathway modulation, and off-target protein changes [65,66]. However, it does not directly measure endosomal escape and remains limited by proteome coverage, dynamic range, and missing measurements. No single technique is sufficient to demonstrate productive intracellular delivery. Robust validation should combine spatial imaging, quantitative cytosolic-delivery assays, LC–MS/MS confirmation of intact degrader in the relevant compartment, and degradation-specific endpoints, including DC_50_, Dmax, and degradation kinetics.

### 4.3. Intracellular Trafficking Versus Productive Ternary Complex Formation

The formation of a productive ternary complex comprising the degrader, target protein, and the E3 ubiquitin ligase (Ubq-l) is the essence of PROTAC. This event can be facilitated or inhibited by intracellular trafficking. The degrader can be released too slowly, from the wrong compartment, or its intracellular distribution is altered, that do not lead to productive target–ligase contacts.

Thus, mechanistic assays should link the release of target molecules inside cells with ternary complex formation, ubiquitination, and proteasome-dependent degradation. Additional options to confirm pathway dependence include competitive target ligands, E3 ligase binders, proteasome inhibitors, lysosome inhibitors, and target protein ubiquitination assays. Structural and mechanistic reviews highlight that successful degradation is not based on target binding, but on induced proximity [67].

### 4.4. Degradation Kinetics

Parameters for degradation potency must be reported as those specific to TPD [68]. Apparent potency is described by half-maximal degradation concentration (DC_50_); whereas, the extent of target depletion is described by maximum degradation (D_max_) [68]. Durability is described by time-to-degradation, residence time, and recovery after washout [69]. As nanocarriers can change local intracellular concentrations, the hook effect—formation of the ternary complex is inversely proportional to concentration—has to be evaluated [69].

A delivery system that lowers DC_50_ but not D_max_ may not be considered superior. Similarly, extended exposure might be due to depot release and not necessarily due to effective degradation [70]. TPD databases and proteomic reviews highlight that to interpret activity, it is crucial to consider activity in the context of degradation-specific metrics such as DC_50_, D_max_, selectivity, and time-dependent impacts [71].

### 4.5. E3 Ligase Expression, Target Abundance, and Cell-Type Selectivity

Cell-type selectivity is weakly linked to the levels of target protein expression and E3 ligase recruited [72]. A degrader that works well in one cell line may not in another due to the E3 ligase being either absent or lowly expressed and/or mutated, or its subcellular location may be changed, preventing it from interacting with the target [73]. Target abundance is also a factor, as more exposure to the intracellular degrader or a longer treatment may be necessary for high amounts of expression.

By comparing previous delivery systems among cell lines, it can be derived that E3 ligase and target expression should be profiled in vitro [74]. E3 ligases can be determined by knockdown/KO/rescue experiments [72], which is particularly important as only a subset of human E3 ligases has been utilized in TPD, yet many E3s are encoded [75].

### 4.6. Programmable Activation

The purpose of the programmable degraders is to limit degradation to specific biological contexts [76]. These can be light, acidic PH, disease-associated enzymes, ROS, hypoxia, microRNA signatures, or tumor-specific metabolites. Conditional degradation: The primary requirement for the mechanism is for the system to be inactive (or at least be very low) without the trigger and to induce target depletion following activation [77].

For instance, DNA-encoded pre-PROTACs controlled by microRNAs (miRNAs) have been reported as a strategy to reach cell-selective and controlled protein degradation [78]. Systems like these need controls to test for trigger specificity, background activation, activation kinetics, and reversibility [77]. Trigger responsiveness must not be tested exclusively in simple chemical buffers.

### 4.7. Safety Mechanisms

Cell viability alone should not be the only indicator of in vitro safety. TPD can cause unanticipated degradation of associated proteins, neosubstrates, or incorporated proteins via non-conformal interactions [79]. Mass spectrometry, transcriptomics, cytokine release assays, mitochondrial stress assays, and immune-cell compatibility can detect early liabilities over the proteome [79]. Proteomic-based tools have proven to be helpful in understanding degradation selectivity, target engagement, mechanism of action, and safety-relevant off-target effects [71].

**Figure 2:**
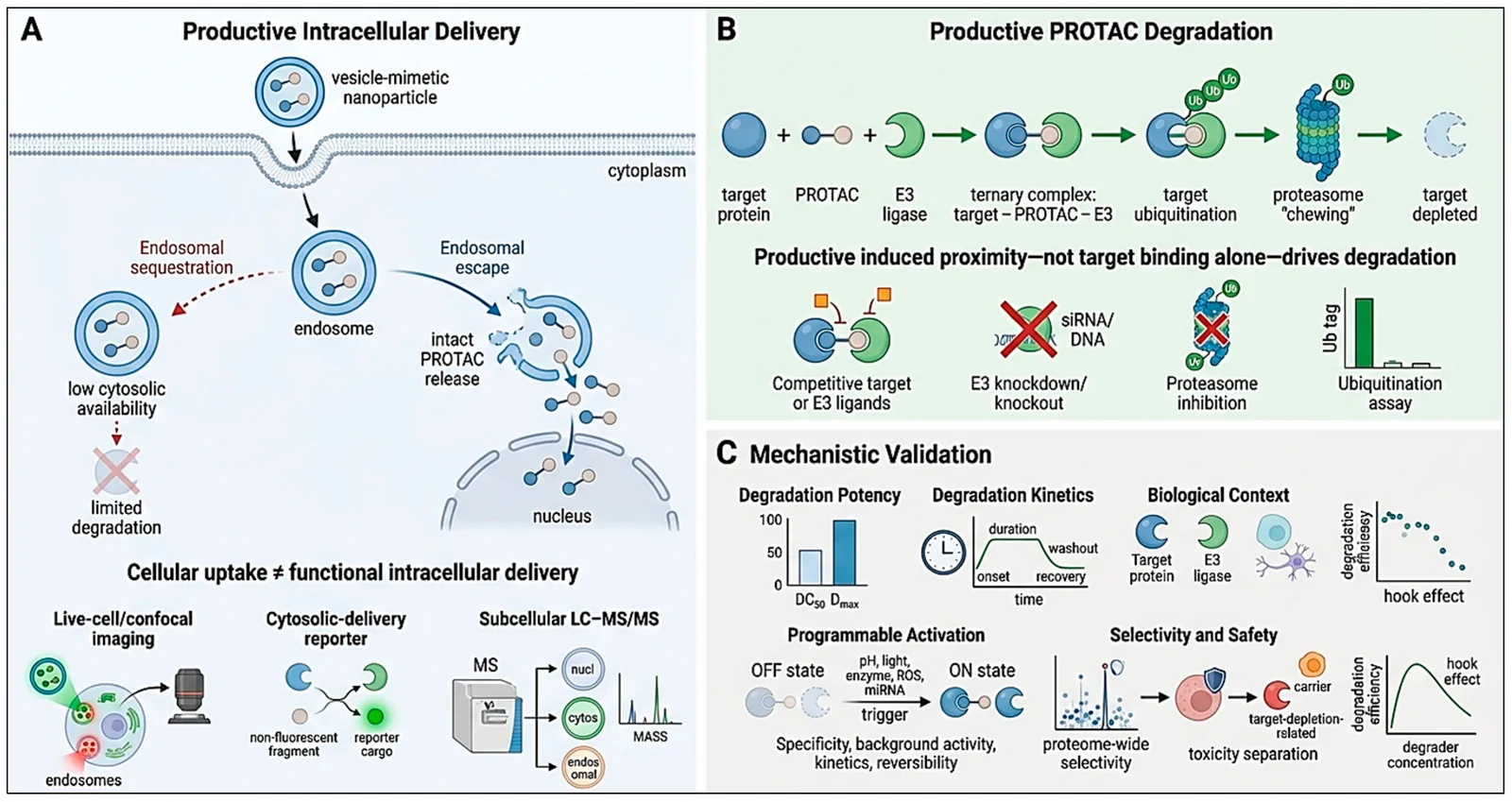
Mechanistic in vitro framework for nano-enabled targeted protein degradation. (**A**) Nanocarrier uptake is followed by either endosomal sequestration or productive escape of an intact PROTAC into the cytosol or nucleus. (**B**) Productive target–PROTAC–E3-ligase complex formation induces ubiquitination, proteasomal degradation, and target depletion. (**C**) Mechanistic validation includes DC_50_, Dmax, degradation kinetics, biological context, programmable activation, proteome-wide selectivity, and safety.

In addition to carrier-free nanoparticles, carrier-driven toxicity, complement activation, inflammatory signaling, and degradation-independent cytotoxicity have to be evaluated during safety testing for nanomedicine-enabled TPD [80,81]. A stringent in vitro package should therefore delineate between three types of toxicity: of the carrier, of the degrader, and that arising from target degradation; this distinction is key before in vivo efficacy studies (Figure 2).

## 5. Functional In Vivo Evidence

In vivo studies it is critical to verify whether target depletion is achieved following nano-degrader delivery with a pharmacologically relevant target-available target [72]. A thorough in vivo investigation combines pharmacokinetics, biodistribution, intracellular delivery, target degradation, pathway modulation, efficacy, and toxicity.

### 5.1. Pharmacokinetics and Biodistribution of Nano-Delivered Degraders

Nanocarriers can modify degrader pharmacokinetics, including apparent solubility, circulation time, interactions among plasma proteins, and distribution in the exposed organ [82]. These effects might enhance efficacy but may also lead to accumulation in clearance organs, including the liver, spleen, and kidney [83]. Thus, it is important to report plasma concentration–time profiles, organ exposure, tumor/target tissue exposure, and recovery of intact degrader via in vivo studies [84]. Carrier design could alter the pharmacokinetic behavior of PROTACs, as improved retention and antitumor activity have been observed in a xenograft model with a PROTAC MZ1 prodrug within lipid nanodisks [27].

### 5.2. Tumor Accumulation Versus Intracellular Bioavailability

The achievement of tumor accumulation cannot be assumed to correlate with degrader availability inside the cell [85]. Nanoparticles can accumulate onto tumor tissue but remain outside cells (extracellular), in stromal areas, inside cells but not in cancer cells (nonmalignant), or endo/lysosomal compartments [86]. For TPD, the active degrader must make it to the subcellular compartment where the target protein and degradation machinery are reachable [87]. Therefore, in vivo studies should be paired with techniques that capture at a cellular resolution, such as immunofluorescence, flow cytometry of dissociated tissue, spatial proteomics, or cell-type-specific degradation assays of the target.

### 5.3. Evidence for Target Degradation in Tissues

One of the main limitations of in vivo degradation studies is assessment by tumor growth inhibition alone, noting the absence of any direct evaluation of target depletion in tissue [88]. If a delivery claim is made, investigators will be expected to demonstrate a decline in target protein levels within excised tumor/affected tissue, downstream pathway modulation of the target, if the target protein has been lost, and protection/reversal/comparator controls if available [89]. The reviews on TPD nanomedicine focus on the need to associate therapeutic benefit to degradation activity that can only be assumed by the formulation efficiency alone.

### 5.4. Dose–Response, Dosing Frequency, and Durability of Degradation

Evaluation of in vivo degradation has to be prioritized over dose and time. One endpoint post-treatment is not enough to characterize a degrader’s pharmacodynamic profile [90]. Research should include the following data: onset of degradation, maximum depletion, duration of depletion, target recovery, and relationship with dosing frequency. Additionally, others are especially significant with regard to TPD that may have long-lasting effects on the PD even after the plasma degrader levels have dropped [90]. Conversely, if the targets are resynthesized very quickly, they need to be administered multiple times despite the enhanced quality of initial degradation [91].

### 5.5. Nanomedicine-Enabled Mitigation of Systemic Toxicity

The ability to achieve stimulus-responsive release or improve tissue selectivity and to limit free degrader exposure will enable us to minimize systemic toxicity in nanomedicine applications [92]. Toxicity mitigation, however, has to be proved, not taken for granted. Endpoints of interest are body weight, hematology, serum biochemistry, cytokine induction, histopathological clearance of organs, immune activation, and comparison to free degrader at matched active exposure [93]. In vivo studies should also be able to differentiate toxicities of the carrier and due to pharmacological target degradation.

### 5.6. In Vivo Validation of Programmable Proximity Platforms

Proof for programmed proximity platforms, and for any other kind of triggers, is crucial to demonstrate that activation is in vivo, under the desired biological or external trigger [94]. Preclinical models with a cell-selective degradation logic via pre-PROTAC systems, such as using microRNAs, have been reported. In vivo components of such a validation include inactive precursor controls, triggered negative controls, spatial control of activation, target degradation in trigger-positive tissue only, and assessment of leakage in the off state [95]. Otherwise, programmable activity cannot be judged as a conclusion deducible from pharmacological experiments but only as a design claim.

### 5.7. Limitations of Current Animal Models and Biomarkers

Human-relevant delivery barriers, immune responses, tumor architecture, E3 ligases, and tissue-specific target biology must often be investigated in an animal model not currently available [96]. Although predominantly used for initial efficacy testing, subcutaneous xenografts may be convenient but may overestimate delivery performance relative to any of the orthotopic, metastatic, immunocompetent, or patient-derived models [97]. Complete translation will need biomarker(s) of exposure, target, downstream pathway modulation, and toxicity. In vivo validation based on tissue-level degradation or mechanism-related pharmacodynamics must be considered as minimum evidence for efficacy endpoints that should not be endorsed when advancing nanoTPD systems into the field [41].

## 6. Translational and Clinical Evidence

The final proof for TPD has to be the clinical translation [98]. The critical issue is Can degradation occur at concentrations that confer sustained pharmacodynamic activity, tolerable safety, and a clear benefit over inhibitors/antibodies or standard-of-care drugs?

### 6.1. Current Clinical Landscape of Targeted Protein Degraders

Within the clinical TPD field, small-molecule degraders, including cereblon (CRBN)-recruiting molecular glues and heterobifunctional PROTACs, are still dominant [99]. Immunomodulatory imide drugs, including thalidomide, lenalidomide, and pomalidomide, are clinically established molecular glue degraders that remodel the substrate recognition ability of CRBN and degrade its neosubstrates, for example, IKZF1 and IKZF3 [100]. More recent clinical candidates are next-generation CRBN modulators and PROTACs primarily targeting oncology [99]. While clinical-stage degradation enables the development of many more degraders in principle, a related review shows that most clinical-stage degraders still target CRBN and a smaller number of others [101].

Androgen receptor (AR) degraders are the most prominent clinical PROTAC examples for prostate cancer; whereas, estrogen receptor (ER) degraders are for breast cancer [102]. ARV-110 and ARV-471 played a pivotal part in the perception that heterobifunctional degraders can advance into human trials, as space as a whole is still in early development when compared to conventional inhibitors and approved molecular glues [103]. Evidence from clinical use therefore favors TPD as a therapeutic modality, although not yet as a widely validated delivery platform.

### 6.2. Why Do Most Clinical Degraders Remain Conventional Small Molecules?

The majority of clinical degraders are still traditional, small molecule candidates because they are appropriate for existing development infrastructure [104]. Compared with multicomponent nanomedicines or externally activated systems, it is much easier to prepare, characterize, prepare a dose of, store, and regulate small compounds [105]. The tendency of molecular glues to be smaller than PROTACs is an attractive property and may be better drug-like candidates [106]. Perhaps molecular glue degraders can leap into clinical use earlier than heterobifunctional PROTACs [107].

Despite their mechanistic benefits, PROTACs face limitations in clinical translation due to size, polarity, conformational flexibility, permeability, solubility, and pharmacokinetic variability [108]. The alterations in oral absorption, intracellular availability, and dose-limiting toxicity may complicate these features [109]. Beyond target engagement, clinical reviews underscore the importance of pharmacokinetics, pharmacodynamics, safety margins, and resistance biology in defining degrader success [110]. However, this ability gives nano-PROTACs a fundamental conflict; while these drug delivery systems address critical liabilities, they introduce new layers of complexity for the manufacture and regulation of new drugs [111].

### 6.3. Translational Barriers for Nano-PROTACs

There are two overlapping translational challenges with Nano-PROTACs [112]. First, they have to meet the typical requirements for nanomedicines: reproducible particle size, polydispersity, surface properties, sterility, endotoxin control, limits on residual solvents, storage stability, and scalable manufacturing [113]. Second, they must meet degrader-specific criteria: degrader integrity preserved, release of active degrader handled, intracellular delivery, and tissue-level target depletion [114].

Small batch academic formulations that have shown antitumor activity in animals do not necessarily mean that they are clinically ready. Scale-up can induce changes in morphology, loading, release kinetics, and biological distribution. Lipid or polymeric systems may be destabilized by sterilization [115]. Particles can alter the aggregation and/or leakage of payloads during long-term storage [116]. Another crucial aspect of TPD is that even minor formulation deviations might lead to varied pharmacodynamic outcomes, as intracellular concentration, ternary complex formation, and target-resynthesis chemo-kinetics reverse degradation [117]. Certain barriers in nano-enabled TPD remain unresolved: bioavailability, safety, delivery efficiency, and off-target effects [118].

### 6.4. Regulatory Complexity of Combination Products and Programmable Nanomedicines

The regulatory pathway for programmable nano-TPD systems will be product-specific and determined by the regulatory status and primary mode of action of their constituent parts, rather than by formulation complexity alone. Under the FDA framework, a product is considered a combination product when it comprises two or more regulated constituent parts, such as a drug, biological product, or device; the primary mode of action determines the lead FDA center and generally informs the premarket pathway [FDA guidance (https://www.fda.gov/regulatory-information/search-fda-guidance-documents/principles-premarket-pathways-combination-products?, accessed on 20 August 2026)]. Thus, a nano-TPD system requiring a dedicated light-, ultrasound-, or magnetic-field-generating device may require coordinated drug–device review; whereas, an internally responsive carrier–degrader formulation is not automatically a combination product. In the European Union, the EMA distinguishes integral, co-packaged, and referenced drug–device configurations and requires device-specific quality information, assessment of the device’s effect on the medicinal product’s quality target product profile and critical quality attributes, and applicable evidence of conformity with the Medical Devices Regulation [EMA guideline (https://www.ema.europa.eu/en/quality-documentation-medicinal-products-when-used-medical-device-scientific-guideline?, accessed on 20 August 2026)].

For nanomaterial-containing drug or biological products, the FDA recommends risk-based, product-specific characterization of composition, particle-size distribution, morphology, surface properties, stability, free versus carrier-associated drug, in vitro release, manufacturing controls, biological fate, biodistribution, and immunogenicity where relevant [FDA guidance (https://www.fda.gov/regulatory-information/search-fda-guidance-documents/drug-products-including-biological-products-contain-nanomaterials-guidance-industry?, accessed on 20 August 2026)] [108]. The EMA similarly emphasizes case-by-case quality, non-clinical, and clinical assessment of nanotechnology-based medicinal products [EMA report (https://www.ema.europa.eu/en/documents/report/nanotechnology-based-medicinal-products-human-use-eu-horizon-scanning-report_en.pdf?, accessed on 20 August 2026)]. For programmable nano-TPD, these principles require critical quality attributes and analytical controls to be linked directly to intact degrader release, intracellular exposure, tissue-level target depletion, and safety. Both inactive and activated product states should be characterized, including off-state leakage, activation thresholds, trigger–response reproducibility, preservation of degrader integrity, and variability in endogenous triggers or external-device output. ICH Q9(R1) provides the applicable lifecycle quality-risk-management framework [ICH guideline (https://database.ich.org/sites/default/files/ICH_Q9%28R1%29_Guideline_Step4_2023_0126.pdf?, accessed on 20 August 2026)], while ICH Q14 and Q2(R2) support the development and validation of quantitative, specific, and stability-indicating release and activation assays [ICH Q14 (https://www.ema.europa.eu/en/ich-q14-analytical-procedure-development-scientific-guideline?utm_source=chatgpt.com, accessed on 20 August 2026), ICH Q2(R2) (https://www.ema.europa.eu/en/ich-q2r2-validation-analytical-procedures-scientific-guideline?, accessed on 20 August 2026)]. Because no dedicated nano-TPD regulatory guidance is currently available, early consultation with the relevant authority will be important for agreeing on product classification, control strategy, and the required non-clinical and clinical evidence.

### 6.5. Clinical Endpoints

Some key endpoints are goal protein decay in tumor/surrogate tissue, downstream pathway modulation, exposure–response relationships, exposure time of decay, and target protein recovery following exposure cessation [119]. The dose range has to be determined by a pharmacodynamic window in which the time to degradation is adequate, and toxicity is tolerable [119].

**Figure 3:**
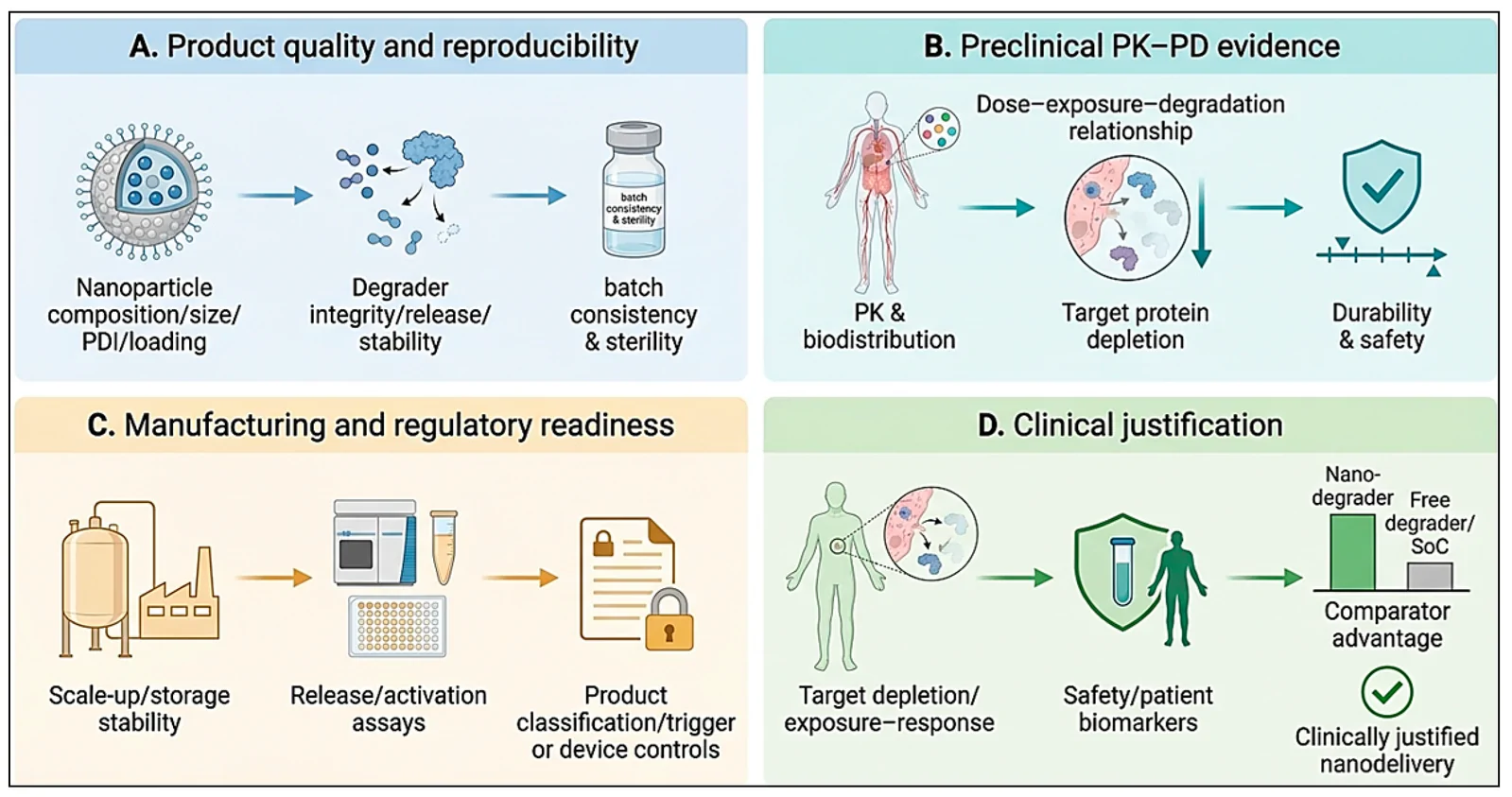
Translational evidence framework for nano-enabled targeted protein degradation. (**A**) Product quality requires reproducible composition, active degrader release, stability, and batch consistency. (**B**) Preclinical evidence should link pharmacokinetics and biodistribution, durability, and safety. (**C**) Translational readiness requires scalable manufacturing, analytical assays, and product-specific regulatory controls. (**D**) Clinical justification depends on exposure–response, target depletion, and demonstrated advantage over the free degrader or standard of care.

An extra endpoint is required for nano-PROTACs: demonstration of enhanced performance of nanodelivery compared with the free degrader or a traditional comparator [120]. However, nanodelivery is still a formulation change and not a clinically justified treatment step up until evidence of improved pharmacokinetics, tissue selectivity, safety, or pharmacodynamic durability is provided (Figure 3, Table 1).

## 7. Critical Comparison of Delivery Platforms

No one delivery platform is ideal for all TPD-associated delivery modalities. The choice of platform varies based on the degrader structure, target tissue, intracellular location, route of administration, and desired degrees of activation control.

### 7.1. Lipid Nanoparticles

For hydrophobic or amphiphilic degraders, lipid nanoparticles are among the most practical systems that can enhance apparent solubility, circulation behavior, and payload protection [121]. Lipid-based systems can also be used to incorporate prodrugs and for stimulus-responsive release [122]. For instance, lipid nanodiscs have enhanced the pharmacokinetic properties and antitumor activity of MZ1 in vivo [27]. They are mainly subject to issues of payload leakage, affinity for clearance organs, inter-batch variability, and a lack of intracellular release [123]. They are most applicable to poorly soluble degraders, with systemic exposure and formulation feasibility being major hurdles.

### 7.2. Polymeric Nanoparticles

Polymeric nanoparticles can be engineered to possess a wide range of degradation rates, surface properties, ligands, and release kinetics [124]. They can be helpful in situations where a sustained release is required for targeting tissues. However, their disadvantages include polymer heterogeneity, residual solvents, slow payload release, and inflammatory response [125]. In the case of TPD, too slow a release can diminish the cell concentration of the compound required for a productive target–ligand interaction [126]. Polymeric systems are best suited for local delivery, depot-like release, or degraders, which have to be exposed for a longer term.

### 7.3. Inorganic Nanocarriers

Inorganic carriers, such as mesoporous silica, gold, iron oxide, and metal–organic framework (MOF) systems, provide structural stability, high loading surface, imaging, and responsiveness to external stimuli [127]. They can be useful in cases where delivery and imaging have to be combined. However, some factors still limit the use of biodegradation, such as long-term retention in tissues, metal-associated toxicity, and regulatory considerations [128]. These are not ideal for almost immediate clinical translation, but are suitable for mechanistic or theranostic TPD research [129].

### 7.4. Biomimetic Vesicles and Cell-Membrane-Coated Systems

Biomimetic vesicles and cell-membrane-coated nanoparticles can be enhanced to improve immune evasion, circulation time, and tropism to disease sites [130]. These systems can be helpful in TPD, where immune clearance or tissue selectivity is a concern; they lack compositional complexity. Membrane source, protein composition, vesicle purity, and batch repeatability are hard to normalize [131]. Such systems may be best suited for diseases in which biological homing offers a good benefit but needs extensive characterization before any translational claims could be made [131].

### 7.5. Antibody-, Aptamer-, and Ligand-Directed Delivery

Delivering to a targeted cell-type by exploiting the specificity of antibodies, aptamers, peptides or small molecule ligands can enhance cell-type selectivity [132]. Among these approaches, antibody-directed degrader conjugates have progressed substantially further than aptamer-, peptide-, or small-molecule-ligand-directed delivery systems. ORM-5029, a HER2-directed conjugate carrying a GSPT1-degrading molecular-glue payload, entered Phase I evaluation in 2022 (NCT05511844 (https://clinicaltrials.gov/study/NCT05511844?, accessed on 20 August 2026)), but its further clinical development was discontinued in 2025. BMS-986497 (ORM-6151), a CD33-directed conjugate carrying a GSPT1 degrader payload, is currently being evaluated in a recruiting Phase I study involving patients with relapsed or refractory acute myeloid leukemia or myelodysplastic syndrome (NCT06419634 trial (https://www.bmsstudyconnect.com/es/en/clinical-trials/NCT06419634.html?, accessed on 20 August 2026)) (ClinicalTrials.gov, 2022, 2024; official ORM-5029 discontinuation announcement (https://www.orumrx.com/news/orum-therapeutics-provides-program-update-and-announces-drug-candidate-nomination?, accessed on 20 August 2026)). These programs provide clinical evidence that antibodies can achieve cell-selective delivery of protein-degrading payloads. However, they should not be described as clinical validation of nanoparticle-based PROTAC delivery, nor should their maturity be extrapolated to aptamer-, peptide-, or small-molecule-ligand-directed systems, which remain preclinical.

### 7.6. Activatable and Self-Assembling Degrader Platforms

The goal of activatable and self-assembling systems is to limit degradation to a particular biological environment. These include light, pH, enzyme, hypoxia, reactive oxygen species (ROS), and miRNA-responsive systems [133]. The notional logic of programmable systems for cell-selective degradation is exemplified by microRNA-triggered DNA-encoded pre-PROTACs [78]. Their drawbacks include validation burden—leakage current, incomplete activation, trigger heterogeneity, and reversibility, aspects that need careful examination [134]. These systems are best suited for high-risk targets where spatial or temporal control is important (Table 2).

### 7.7. Translational Maturity and Clinical Readiness of Delivery Platforms

Evidence supporting delivery-enabled targeted protein degradation remains predominantly preclinical. Lipid nanodisks improved the systemic persistence, tumor penetration and xenograft efficacy of an MZ1 prodrug (Pan et al., 2025) [27]; whereas, polymeric nano-PROTACs achieved intratumoral BRD4 or CDK4/6 depletion and tumor suppression in mouse models (Gao et al., 2022; Yang et al., 2024) [24,138]. Gold-nanoparticle-based multi-headed PROTACs and genetically engineered lysosome-targeting exosomes remain cellular proof-of-concept systems and represent nanoparticle-assembled or exosome-mediated degradation rather than clinically validated PROTAC formulations (Wang et al., 2020; Wang et al., 2023a) [139,140]. Aptamer-directed, activatable and self-assembling systems have demonstrated target degradation and antitumour activity in animal models (He et al., 2021; Gao et al., 2024; Wang et al., 2023b) [76,141,142]. Antibody–degrader conjugates are the only delivery class considered here to have entered Phase I evaluation; however, the clinical candidates deliver GSPT1 molecular-glue degraders rather than nanoparticle-formulated, bifunctional PROTACs. ORM-5029 entered Phase I but was subsequently terminated; whereas, BMS-986497 remains under Phase I investigation ([143]; NCT05511844 (https://clinicaltrials.gov/study/NCT05511844, accessed on 20 August 2026); NCT06419634 (https://clinicaltrials.gov/study/NCT06419634, accessed on 20 August 2026)). Therefore, translational maturity should be assigned to each complete carrier–degrader product rather than inferred from the clinical precedent of the carrier class alone. Accordingly, the highest documented development stage, direct supporting evidence, and principal translational barriers for each delivery platform are summarized in Table 3.

### 7.8. Quantitative Performance and Limitations of Cross-Platform Comparison

Quantitative comparison of delivery-enabled targeted protein degradation systems remains challenging because published studies employ heterogeneous targets, degrader chemistries, biological models, doses, treatment schedules, comparators, and analytical endpoints [41,142]. Reported measurements include DC_50_ and Dmax values in cultured cells, tumor-tissue target depletion, intratumoral degrader concentration, apoptosis, tumor-growth inhibition, and survival [41,138]. These endpoints describe different stages of the delivery–degradation–response pathway and therefore should not be treated as interchangeable measures of platform performance. Table 4 consequently presents author-reported values without normalization or numerical ranking. For the representative systems summarized here, no study has directly compared different carrier classes using the same degrader, target, model, dose, exposure, and analytical workflow.

Nevertheless, individual studies demonstrate quantitatively measurable delivery advantages. Lipid-nanodisk-displayed MZ1 prodrug produced an antitumor effect that was nearly comparable to free MZ1 administered at a tenfold higher dose in the same xenograft study [27]. In an MDA-MB-231 xenograft model, PGD7 POLY-PROTAC nanoparticles administered at 10 mg kg^−1^ ARV771-equivalent suppressed tumor BRD4 expression by approximately 80% and delayed tumor growth by approximately 50%; whereas, free ARV771 produced negligible effects at the matched dose [138]. The bioorthogonal variant of this platform increased intratumoral ARV771 concentration 3.9-fold relative to free ARV771 at 36 h, although this exposure measurement does not itself establish equivalent enhancement of target degradation.

Target-directed systems have also generated quantitative cellular degradation data. An aptamer–PROTAC conjugate produced BRD4 degradation with a DC_50_ of 22 nM and D_max_ greater than 90% in MCF-7 cells, compared with 13 nM and greater than 90%, respectively, for the unconjugated parental PROTAC [142]. Thus, aptamer conjugation preserved high degradation efficiency but did not improve intrinsic cellular degradation potency in this model; its principal advantage was tumor-cell targeting. More recently, the PSMA-responsive, in situ self-assembling Psa-AR system produced approximately 80% full-length androgen-receptor depletion, 74% AR-V7 depletion, and 65% HSP90 depletion in 22Rv1 cells. In xenografts, it achieved up to 78% tumor-growth inhibition and extended median survival by 15 days relative to combined enzalutamide and pimitespib treatment [41].

## 8. Future Directions

Next-generation TPD delivery should go beyond enhancing solubility or circulation time.

### 8.1. Design of Delivery Systems Around Degradation Biology

The degradation mechanism must serve as a basis for a delivery system. In the case of proteasome-directed degraders, the carrier needs to enable access to the cytosol or nuclei [145]. For lysosome-directed delivery, endosomal/lysosomal transport can be advantageous and not constraining [146]. This distinction should be considered in terms of carrier selection, release kinetics, and route of administration [145]. Future research efforts should then identify the biological bottleneck and, based on this, choose the formulation platform.

### 8.2. Tissue-Selective E3 Ligase Recruitment

A few E3 ligases are used by most existing degraders, notably CRBN and von Hippel–Lindau (VHL) [147]. This reduces target organ specificity and can raise systemic toxicity levels. E3 ligases that are enriched in tissue, diseases, and/or compartments should be used for future degradation design [147]. Much of the E3 ligase family still holds many opportunities for targeting with ligands [75].

### 8.3. Multi-Target and Sequential Degradation Systems

Adaptive diseases like cancer, inflammation, and neurodegeneration may require single-target degradation. Degraders could be used individually to target multiple players, or in combination in a co-delivery or sequential degradation platform that could dampen compensatory signaling [148]. However, they cannot be left uncontrolled, as a more widespread degradation can enhance toxicity [149]. In future work, situations where multitarget degradation is biologically warranted and those in which such degradation would risk unnecessary pharmacological implications have to be clearly demarcated.

### 8.4. AI-Guided Degradation–Carrier Codesign

Artificial intelligence (AI) may help in the co-surgical advance of degraders and carriers, in which molecular properties, linker architecture, carrier composition, release behavior, cell uptake, and degradation pharmacodynamics are integrated [150]. The major benefit of AI will certainly not remain as a straightforward prediction of potency alone; instead, it lies in prioritizing designs that resolve the balance among degradability, deliverability, safety and security, and manufacturability [151]. Degrader activity data from curated datasets like TPDdb can be employed to standardize the data used for model development [70].

### 8.5. Minimal Criteria for Claiming Translational Relevance

Fulfilling only a minimum set of evidence requirements is a prerequisite for a nano-enabled or programmable TPD system to be viewed as translationally relevant: reproducible composition, intact release of the active degrader, degradation of target in relevant cells, and feasibility of manufacturing [151]. In programmable systems, authors should also demonstrate low off-state activity, trigger-specific activation, and reversibility in case it is pointed out as such [152]. These criteria are essential to render the formulation a reasonable therapeutic candidate; otherwise, it is a mechanistic prototype.

## 9. Conclusions

The availability of TPD tools is no longer restricted by design: it is now being determined by delivery, biodistribution, cell entry, and even pharmacodynamic control. Nanomedicine and programmable proximity platforms can overcome these barriers, but only with strict criteria of reproducible formulation, intact drug release, target depletion in relevant tissues, long-lasting pharmacodynamic readout, safety, and superiority over a free degrader or conventional inhibitor. Moving forward, for each new degrader, we should plan for including degrader chemistry, carrier design, disease biology, and clinical biomarkers at the ground level. With these in hand, delivery can be considered not as an add-on to a therapeutic protein formulation but as an outcome design principle that is critical to achieving clinically reliable therapeutic protein degradation agents.

## Figures and Tables

**Table 1:** Translational evidence framework for delivery-enabled targeted protein degradation-based therapeutics.

Evidence Domain	Minimum Evidence Required	Why It Matters	Common Weakness	Translational Implication	Key References
Composition	Size, charge, morphology, loading, and stability	Defines product identity	Single-batch reporting	Weak reproducibility	[15,16]
Degrader integrity	Intact active degrader after loading/releasing	Confirms functional payload	Total drug measured only	Uncertain pharmacology	[27]
Cellular delivery	Uptake plus intracellular bioavailability	Separates entry from activity	Uptake used as a proxy	Overestimated efficacy	[16]
Mechanism	Target depletion, E3 dependence, ubiquitination	Confirms the TPD mechanism	Viability used as a proxy	Mechanistic ambiguity	[67]
Selectivity	Proteome-level off-target analysis	Definition of degradation specificity	Single-target assay only	Safety uncertainty	[71]
In vivo exposure	Pharmacokinetics, biodistribution, tissue exposure	Links dose to delivery	Tumor volume reported alone	Weak translational claim	[27]
Tissue degradation	Target depletion in diseased tissue	Confirms in vivo TPD action	Efficacy without tissue PD	Cannot prove mechanism	[16]
Programmability	Trigger specificity, off-state leakage, and reversibility	Validates controlled degradation	Trigger tested only in the buffer	Poor clinical confidence	[16,19]
Clinical readiness	Manufacturing, storage, biomarkers, comparator benefit	Supports translation	No free-degrader comparison	Limited development value	[101]

**Table 2:** Comparative analysis of delivery methods of targeted protein degradation therapeutics.

Platform	Typical Design	Main Rationale	Main Limitation	Best Use	Ref
Lipid systems	LNPs, lipid nanodisks and lipid–PROTAC prodrugs	Solubilization; prolonged exposure	Leakage; hepatic/splenic uptake	Poorly soluble systemic PROTACs	[27]
Polymeric systems	PLGA, PEGylated polymers, block copolymers	Tunable release; surface engineering	Polymer heterogeneity; scale-up variability	Sustained or local delivery	[15]
Inorganic carriers	Silica, MOFs, gold, iron oxide, ZIF-8	High loading; imaging/triggered release	Persistence; toxicity concerns	Theranostic or triggered TPD	[135]
Biomimetic systems	EVs, membrane-coated nanoparticles	Immune evasion; biological tropism	Source variability; complex QC	Homing-driven delivery	[136]
Targeted systems	Antibody-, aptamer-, peptide-, and ligand-directed carriers	Cell selectivity	Receptor heterogeneity; endosomal trapping	Receptor-positive disease	[137]
Activatable systems	Light-, pH-, enzyme-, ROS-, hypoxia-, miRNA-responsive designs	Conditional degradation	Leakage; trigger heterogeneity	High-risk targets needing control	[19]
Carrier-free systems	Amphiphilic degraders, prodrug nanoassemblies	High loading; fewer excipients	Premature disassembly; limited precedent	Excipient-sparing delivery	[30]

**Table 3:** Comparative translational maturity of delivery platforms for targeted protein degraders.

Delivery Platform	Highest Documented Stage	Direct Evidence	Principal Translational Gap	Ref
Lipid nanodisks	In vivo preclinical	MZ1-prodrug PK and xenograft efficacy	CMC; repeat-dose PK–PD and safety	[27]
Polymeric nanoparticles	In vivo preclinical	Tumor BRD4/CDK4/6 depletion	Batch control; GLP safety	[24]
Inorganic nanoparticles	Cellular proof-of-concept	Gold-NP-mediated ALK degradation	In vivo PK, clearance and safety	[139]
Biomimetic/exosomal systems	Cellular proof-of-concept	LYTEX-mediated membrane-protein degradation	Source/QC, potency and immunogenicity	[140]
Aptamer–PROTAC conjugates	In vivo preclinical	Tumor-selective degradation and efficacy	Stability, penetration and receptor heterogeneity	[142]
Antibody–degrader conjugates	Phase I; no mature outcomes	ORM-5029 terminated; BMS-986497 recruiting	Human safety/efficacy and antigen heterogeneity	[143] NCT05511844 (https://clinicaltrials.gov/study/NCT05511844, accessed on 20 August 2026); NCT06419634 (https://clinicaltrials.gov/study/NCT06419634, accessed on 20 August 2026)
Activatable nano-PROTACs	In vivo preclinical	Triggered intratumoral BRD4 depletion	Off-state leakage; trigger reproducibility	[76]
Self-assembling nano-PROTACs	In vivo preclinical	NIR-controlled degradation in tumor-bearing mice	Colloidal stability, scale-up and repeat-dose safety	[141]

**Table 4:** Representative quantitative outcomes reported for delivery-enabled targeted protein degradation platforms.

Platform/System	Model and Conditions	Principal Quantitative Outcome	Interpretation and Limitation	Ref
Lipid nanodisk–MZ1 prodrug, LND-MZ1	Breast-cancer xenograft; LND-MZ1 at 2 mg kg^−1^	Antitumor effect nearly comparable to free MZ1 at 20 mg kg^−1^ under the same schedule	Within-study tenfold dose comparison; not an exposure-matched carrier comparison	[27]
PGD7 POLY-PROTAC nanoparticles	MDA-MB-231 xenografts; intravenous 10 mg kg^−1^ ARV771-equivalent, every 3 days for five administrations	Approximately 80% tumor BRD4 suppression and approximately 50% tumor-growth delay; matched free ARV771 had negligible activity	Provides tumor-tissue pharmacodynamic and efficacy data; small preclinical cohort	[138]
Sequential-responsive PSRN	CT26 cells and xenografts	Approximately 2.6- and 3.0-fold greater in vivo CDK4 and CDK6 degradation than free PROTAC after single intravenous administration	One polymer degrader system	[24]
Bioorthogonal POLY-PROTAC system	MDA-MB-231 xenografts; PED pretargeting followed by N_3_@PGDA7	At 36 h, intratumoral ARV771 was 3.9-fold higher than free ARV771 and 1.9-fold higher than N_3_@PGDA7 without pretargeting	HPLC-measured drug exposure, not a direct measurement of BRD4 depletion	[138]
Gold-nanoparticle multi-headed degrader, Cer/Pom-PEG@GNP	EML4–ALK-positive NCI-H2228 cells	Approximately 76.2% EML4–ALK depletion after 12 h; cell-viability IC_50_ of 4.8 µM	Figure-derived in vitro values; the construct uses the nanoparticle surface as a multivalent degrader scaffold, and no in vivo efficacy was reported	[139]
Gold-nanocluster hybrid PROTAC, GNCTAC	HER2-positive SKBR3 cells	>95% HER2 degradation; effect sustained for at least 72 h	no definitive in vivo translational validation	[141]
Cancer-cell-membrane-coated PIPD nanoparticle, CM8988-PIPD	PATU-8988 and PL-45 pancreatic-cancer cells	Mean particle size approximately 124.8 nm; apoptosis exceeded 50% in both cell lines	Apoptosis is a functional endpoint rather than a quantitative degradation parameter; study was restricted to in vitro evaluation	[144]
Aptamer–PROTAC conjugate, APR	Nucleolin-positive MCF-7 cells	BRD4 DC_50_ = 22 nM and D_max_ > 90%; parental PROTAC DC_50_ = 13 nM and D_max_ > 90%	Non-nanoparticulate targeted conjugate; targeting advantage did not increase intrinsic cellular degradation potency	[142]
Region-confined ROS/hypoxia-activatable PGDAT@N	MDA-MB-231 and HN30 xenografts with 671 nm irradiation	Tumor disappearance during observation in 4/6 MDA-MB-231 and 5/6 HN30 mice, versus 1/6 and 2/6, respectively, with PGDAT plus irradiation	Multicomponent PROTAC–photodynamic treatment; tumor response cannot be attributed solely to BRD4 degradation, and disappearance does not establish permanent cure	[76]
In situ self-assembling Psa-AR nano-PROTAC	22Rv1 cells and xenografts	Cellular depletion: AR 80%, AR-V7 74%, and HSP90 65%; up to 78% tumor-growth inhibition and median-survival extension of 15 days versus combined inhibitors	Cellular degradation percentages and animal efficacy are distinct endpoints; PSMA expression is required for selective assembly and uptake	[41]

## Data Availability

No new data were created or analyzed in this study. Data sharing is not applicable.
